# Transforming Growth Factor *β* Signaling Upregulates the Expression of Human GDP-Fucose Transporter by Activating Transcription Factor Sp1

**DOI:** 10.1371/journal.pone.0074424

**Published:** 2013-09-12

**Authors:** Yu-Xin Xu, Anna Ma, Li Liu

**Affiliations:** 1 Department of Molecular and Cell Biology, Boston University Goldman School of Dental Medicine, Boston, Massachusetts, United States of America; 2 Center for Human Genetic Research and Cardiovascular Research Center, Massachusetts General Hospital, Boston, Massachusetts, United States of America; University of Massachusetts Medical, United States of America

## Abstract

GDP-fucose transporter plays a crucial role in fucosylation of glycoproteins by providing activated fucose donor, GDP-fucose, for fucosyltransferases in the lumen of the Golgi apparatus. Fucose-containing glycans are involved in many biological processes, which are essential for growth and development. Mutations in the GDP-fucose transporter gene cause leukocyte adhesion deficiency syndrome II, a disease characterized by slow growth, mental retardation and immunodeficiency. However, no information is available regarding its transcriptional regulation. Here, by using human cells, we show that TGF-β1 specifically induces the GDP-fucose transporter expression, but not other transporters tested such as CMP-sialic acid transporter, suggesting a diversity of regulatory pathways for the expression of these transporters. The regulatory elements that are responsive to the TGF-β1 stimulation are present in the region between bp −330 and −268 in the GDP-fucose transporter promoter. We found that this region contains two identical octamer GC-rich motifs (GGGGCGTG) that were demonstrated to be essential for the transporter expression. We also show that the transcription factor Sp1 specifically binds to the GC-rich motifs *in vitro* and Sp1 coupled with phospho-Smad2 is associated with the promoter region covering the Sp1-binding motifs *in vivo* using chromatin immunoprecipitation (ChIP) assays. In addition, we further confirmed that Sp1 is essential for the GDP-fucose transporter expression stimulated by TGF-β1 using a luciferase reporter system. These results highlight the role of TGF-β signaling in regulation of the GDP-fucose transporter expression *via* activating Sp1. This is the first transcriptional study for any nucleotide sugar transporters that have been identified so far. Notably, TGF-β1 receptor itself is known to be modified by fucosylation. Given the essential role of GDP-fucose transporter in fucosylation, the finding that TGF-β1 stimulates the expression of this transporter, suggests a possible intracellular link between the function of nucleotide sugar transporter and the TGF-β signaling pathway.

## Introduction

Most secreted and membrane proteins, including ion channels, blood groups, growth factors, and their receptors in eukaryotes, are glycosylated in the lumen of the Golgi apparatus. These glycoproteins are modified with a variety of sugars including fucose, galactose, *N*-acetylglucosamine, *N*-acetylgalactosamine and sialic acid. The sugar modifications play key roles in development of multicellular organisms and the aberrant modifications are linked to many human diseases[1]–[3]. Glycosylation requires activated sugar donors, nucleotide sugars, which are synthesized in cytosol in mammals and must be transported into the lumen of the Golgi apparatus where they serve as substrates for glycosyltransferases. The translocation of the nucleotide sugar substrates from cytosol to the lumen of the Golgi apparatus is carried out by specific nucleotide sugar transporters (NSTs). NSTs are structurally conserved, hydrophobic, and type III multi-transmembrane proteins mainly present on the membrane of the Golgi apparatus in all eukaryotic organisms [4], [5]. A specific NST may transport, in a saturable manner, one or multiple nucleotide sugar substrates. NSTs are also antiporters with their corresponding nucleoside monophosphates. This antiport system provides energy for the transport of nucleotide sugars into the Golgi apparatus lumen. In humans and *Caenorhabditis elegans*, there are seven sugars in glycoconjugates, but 17–18 putative NSTs are predicted from their genomic databases [6], [7]. This indicates that NSTs possess redundant functions by transporting overlapping substrates. The functional redundancy has been experimentally validated in *C. elegans* [8].

Given their essential function in glycosylation, NSTs have been shown to play critical roles in development and organogenesis of mammals [2], [6], [7]. Mutations in NST genes cause human diseases. For example, leukocyte adhesion deficiency syndrome II (LAD II) is caused by mutations in the GDP-fucose transporter gene [9]–[11] and Schneckenbecken dysplasia is resulted from mutations in a UDP-glucuronic acid/UDP-*N*-acetylgalactosamine (UDP-GlcA/GalNAc) dual substrate transporter gene [12]. Functional deficiency of NSTs also results in developmental defects in cattle e.g., complex vertebral malformation [13]. Consistent with the roles of NSTs in growth and development, we recently showed that NSTs are intimately involved in protein synthesis and secretion. Down-regulation of NST gene expression in human cells resulted in global defects in protein synthesis and secretion of not only glycoproteins, but also non-glycoproteins [14].

Little is known about the NST gene expression and transcriptional regulation. Human tissue-specific expression profile study revealed that NSTs are ubiquitously expressed in both adult and fetal tissues and their expression levels are linked to the development of human diseases [15]. For example, UDP-galactose transporter is overexpressed in colon cancer [16]. However, information, regarding extracellular stimuli, intracellular signaling pathway and transcription factor(s) that regulate the gene expression of NSTs, has been lacking.

TGF-β signaling pathway regulates a variety of cellular biological processes such as cell migration, adhesion, proliferation and differentiation. Many of these events result from the expression regulation of key target genes. The TGF-β signal transduction involves a family of membrane receptor protein kinases (TGF-β receptor I and II) and a family of receptor substrates such as the R-Smad proteins (including Smad2, Smad3 and Smad4). Transcriptional regulation *via* TGF-β family signaling is a dynamic process. Following TGF-β induction, Smad2 and/or Smad3 are phosphorylated by the activated TGF-β receptors and then dissociated from receptors to form Smad complexes with Smad4, which are subsequently translocated into nucleus [17], [18], where they functionally cooperate with other promoter-specific transcription factors and regulate TGF-β-induced transcription of target genes depending on a TGF-β stimulus and the corresponding cellular response in a given cell type and at a given time.

In this study, we demonstrate that TGF-β1 specifically stimulates the expression of GDP-fucose transporter, but not CMP-sialic acid and UDP-GlcA/GalNAc transporters, suggesting a diversity of regulatory pathways for the expression of these transporters. We found that the GDP-fucose transporter promoter contains two identical octamer GC-rich motifs that are responsive to the TGF-β1 stimulation. We provide evidence that the transcription factor Sp1 specifically binds to the two GC-rich motifs, and mutations in either motif abolished the Sp1 binding. Using chromatin immunoprecipitation (ChIP), we show that Sp1 along with the phosphorylated Smad2 (pSmad2) are specifically associated with the GDP-fucose transporter promoter upon the induction of TGF-β1. Using a luciferase reporter system, we show that TGF-β1-mediated gene expression of GDP-fucose transporter is Sp1 dependent. This is the first study on transcriptional regulation of NSTs in any organisms in which NSTs have ever been identified. Notably, TGF-β1 receptor itself is modified by fucosylation [19]; it is therefore possible that the transcriptional regulation of this transporter by TGF-β1 may provide a feedback for the TGF-β signaling pathway.

## Materials and Methods

### Oligonucleotide Sequences

Oligonucleotide sequences for luciferase constructs containing various length of the upstream sequences of the GDP-fucose transporter gene (NP_060859). Forward upstream primers: Up1316∶5′-TCCCCGCACGCACTTCTGGAAG-3′; Up1097∶5′-CCGGCGAGGCTGGCAAGGTG-3′; Up919∶5′-GGAGCTGATGCGGCTGGACC-3′; Up681∶5′-CCACAACATTGTTATGGAAG-3′; Up626∶5′-AACGCGAGTCTCCGGAGGTG-3′; Up525∶5′-GTCGGGGGACGGAGGCTCCG-3′; Up330∶5′-TTTAAGGGCAAGGCGGGGCGTG-3′; Up268∶5′-GTCCAGGGCCCGCCTCCCGG-3′; Up139∶5′-AAGCCCCGAGCCCCTCTGACC-3′; backward downstream primer: 5′-GTGCAGGATCCTGGACCGCTTC-3′. The PCR fragments were subcloned into pGL3 basic vector at Bgl II/Hind III and Nco I sites. All the primer sequences for RT-PCR were derived from exons of the corresponding genes. RT-PCR and qRT-PCR primers for the GDP-fucose transporter, forward primer-1 (RT-PCR): 5′-ACCTCCATCTCCATGGTGTTC-3′ (from exon 2); backward primer-1 (RT-PCR): 5′-GCTCGTCCACCAGAGGAAGC-3′ (from exon 3); forward primer-2 (qRT-PCR): 5′-CTGCTGCTCAAGCAGACCAC-3′ (from exon 2); backward primer-2 (qRT-PCR): 5′-CAGCACGCCGAAGACGGTGC-3′ (from exon 3). RT-PCR primers for the CMP-sialic acid transporter (NP_006407), forward primer: 5′-GGGTTTGGCGCTATAGCTATTG-3′ (from exon 5); backward primer: 5′-GAGTACCCAGGGCAAAGGTG-3′ (from exon 8). RT-PCR primers for UDP-GlcA/GalNAc transporter (NP-055954), forward primer: 5′-GGGTGGGAAAGGCGCTCAGAG-3′ (from exon 3); backward primer: 5′-GCAATGGCCAGGGTGGGCAG-3′ (from exon 8). Primers for ChIP assay of the GDP-fucose transporter promoter region: forward primer-1 (ChIP PCR): 5′-AACGCGAGTCTCCGGAGGTG-3′, forward primer-2 (ChIP qPCR), 5′-GAACTCCGCCTCTCTGGGGC-3′; backward primer (ChIP PCR/qPCR): 5′-CGGACATCCGAGGCCGACTC-3′. RT-PCR and qRT-PCR primers for β-actin, forward primer-1∶5′-GCTTCACCACCACGGCCGAG-3′ (from exon 5); backward primer-1 (RT-PCR): 5′-CAGCGAGGCCAGGATGGAGC-3′ (from exon 7); forward primer-2 (qRT-PCR): 5′-GAAGAGCTACGAGCTGCCTGA-3′ (from exon 5); backward primer-2 (for qRT-PCR): 5′-CATGATGGAGTTGAAGGTAG-3′ (from exon 6).

### Cell Culture, TGF-β1 Induction, Whole Cell Lysates, and Western Blotting

**Figure 1:**
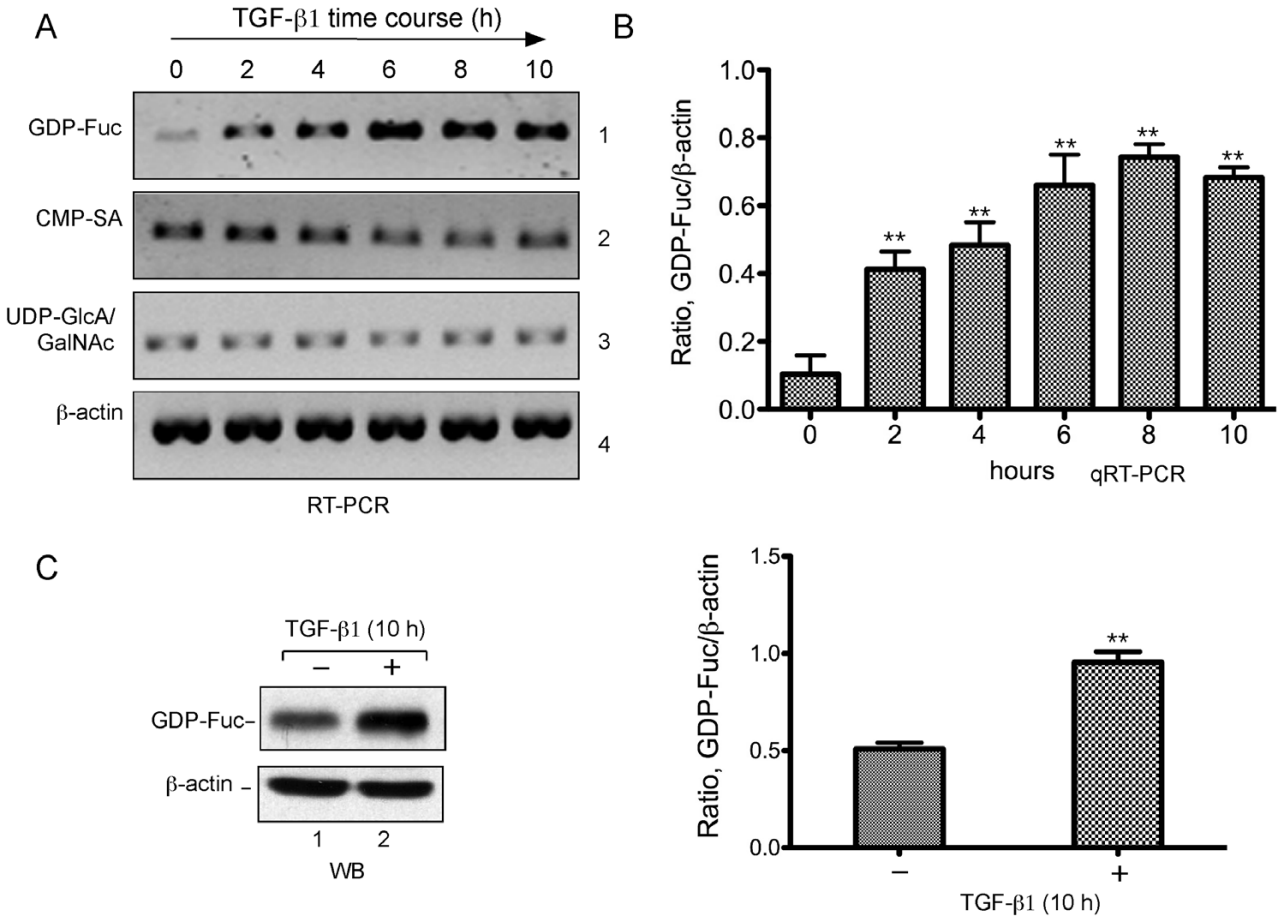
TGF-β1 stimulates the GDP-fucose transporter expression. HeLa cells were serum-starved, then cultured in presence of human recombinant TGF-β1 and harvested over the time course during TGF-β1 induction. **A**. RT-PCR analyses of NST expression. Total RNA was isolated from the cells treated with TGF-β1 at the indicated times (top) and reverse transcribed. RT-PCR was carried with the primers specific to the GDP-fucose (GDP-Fuc), CMP-sialic acid (CMP-SA) and UDP-GlcA/GalNAc transporters as well as β-actin (panels 1–4, respectively). PCR products were analyzed on a 1% agarose gel. **B**. qRT-PCR analyses of NST expression. Experiment with the similar time course upon TGF-β1 induction was performed but with three replicates (see Materials and Methods for details). Total RNA and cDNA were obtained as in A. qRT-PCR was carried out with the primers for GDP-Fuc and β-actin. Error bars represent standard deviation from three replicates. P values were obtained with t-test as compared with time 0. **, P<0.01. **C**. Western analysis of GDP-fucose transporter expression. Total cell lysates were prepared from the cells treated without (lane 1) or with (lane 2) TGF-β1 for 10 h. Blot was probed with antibodies against GDP-fucose transporter (top) and β-actin (bottom) (left panel). Quantification of GDP-fucose transporter expression (right panel) was obtained from densitometric analyses of three Western blots as in left panel. **, p<0.01 (t-test).

HeLa and HEK293 cells were cultured in DMEM medium (Invitrogen) supplemented with 10% fetal bovine serum (FBS) (Atlanta Biologicals). For a time course TGF-β1 induction, HeLa cells were inoculated in 4 wells (4 replicates) in a 6-well plate for each time point. At about 60% confluence, cells were incubated in the 0.5% FBS in DMEM for 24 h, then human recombinant TGF-β1 (10 ng/ml) (Invitrogen) was added for various times as indicated in Fig. 1. Cells were then harvested for total RNA or whole cell lysate. Cells from one of 4 wells at each time point were counted. Whole cell lysates were prepared by solubilizing cells in a lysis buffer [0.5% Triton X-100, 0.5% sodium deoxycholate, 50 mM Tris-HCl (pH 7.5), 250 mM NaCl, 5 mM EDTA and 50 mM sodium fluoride] in presence of complete protease inhibitor cocktail (Roche). Western blotting was carried out with 10% SDS-PAGE and blots were probed with rabbit anti-GDP-fucose transporter, which was generated from this laboratory [14], anti-β-actin, -cdc2 or -Sp1 (Santa Cruz) antibodies.

### siRNA Transfection

siRNAs for Sp1 (5′-GAGCCACUCAGUCUCUUCC-3′) and a negative control were obtained from Qiagen. Transfection was carried out as previously described [14], [20]. Briefly, cells (∼3×10^5^) were grown in DMEM medium in a 6-well plate described above. At about 30% confluence, siRNAs were transfected into the cells with lipofectamine 2000 according to the manufacturer’s procedure (Invitrogen). Transfected cells were harvested ∼96 h after transfection for total RNA or whole cell lysate.

### Total RNA Extraction, End-point PCR and Quantitative Real Time PCR/RT-PCR (qPCR/qRT-PCR)

Total RNA extraction was achieved with TRIzol (Invitrogen) followed by RQ1 DNase I (RNase free) (Promega) treatment according to the manufacturer’s instructions. cDNA was obtained using the SuperScript first-strand synthesis system (Invitrogen). End-point PCR and RT-PCR amplification was carried out with high fidelity PCR system (Roche) and BIO-X-ACT™ Short Mix containing DNA polymerase (Bioline), respectively. qPCR/qRT-PCR products were obtained from an ABI Prism 7000 Sequence Detection System (Applied Biosystems) using a qPCR/qRT-PCR reaction mixture containing 50 ng of precipitated DNA or cDNA, 5 pmol/each primer (sequences detailed in Oligonucleotide sequences**)** and SYBR Green PCR master mix (Applied Biosystems). The results were analyzed with an ABI Prism 7000 SDS software (Applied Biosystems).

### Luciferase Reporter Constructs and Luciferase Activity Assay

Various upstream sequences of the GDP-fucose transporter promoter region were achieved by PCR amplifications using 5′ end serial deletion primers and a 3′ end primer next to the start codon of luciferase, and subcloned into pGL3 basic vector (Promega) at Bgl II/Hind III and Nco I sites. The constructs were transfected into HEK293 cells with lipofectamine 2000 as described in siRNA transfection. After 24 h, whole cell lysates were prepared and analyzed using luciferase assay system (Promega) according to the manufacturer’s procedure. Luciferase activity was measured on a Veritas™ Microplate Luminometer (Terner BioSystems).

### Gel Mobility Shift Assay

Nuclear extract was prepared from HeLa cells as described [21]. Single strand wild type (WT) or mutant sense and antisense oligonucleotides covering GC-rich motifs were synthesized from Eurofins MWG Operon. Double strand oligonucleotides were obtained by annealing equal micromolars of single strand sense and antisense oligonucleotides at 95°C for 5 min and then cooling down to 25°C for 10 min. The double strand oligonucleotides were end-labeled with [γ^32^P] ATP (PerkinElmer) using T4 polynucleotide kinase (New England Biolabs). Labeled oligonucleotides were purified using DNA-binding column (Roche) for subsequent binding reactions. Incubation of the labeled oligonucleotides with nuclear extract was carried out in a binding buffer (60 mM KCL, 6 mM MgCl_2_, 12 mM Hepes, pH 7.9, 36% glycerol, 6% polyvinyl alcochol) in presence of poly [d(I-C)] (0.1 µg/µl) at 30°C for 20 min followed by incubation of anti-Sp1, -ATF1 or -c-Jun antibodies (Santa Cruz) at 30°C for 10 min. The reaction mixtures were analyzed on a 4% native polyacrylamide gel and autoradiography.

### ChIP Assay

ChIP extract preparation and assay were performed as previously described [20], [22], [23] with minor modifications. Briefly, HeLa cells were serum-starved in DMEM medium with 0.5% FBS for 24 h, and then incubated with or without the recombinant TGF-β1 (10 ng/ml) for 12 h. For the siRNA-based ChIP, HeLa cells were transfected with the control or Sp1-specific siRNAs. After 48 h, the cells were serum-starved and stimulated with TGF-β1 as described above. The precipitated DNA was used for PCR and qPCR reactions.

## Results

**TGF-**β**1 Stimulates the Expression of GDP-fucose Transporter**

Previous studies show that the expression of NSTs is dynamically regulated during development [15]. However, the transcription regulation of NSTs such as extracellular signal, transcription factor(s) and promoter elements is largely unknown. To investigate this, we selected GDP-fucose transporter (SLC35C1) due to its implication in human disease. We first used various growth factors including TGF-β1, insulin, or interferon-γ for up- or down-regulation of the GDP-fucose transporter expression together with CMP-sialic acid (SLC35A1) and UDP-GlcA/GalNAc (SLC35D1) transporters. Serum-starved HeLa cells were incubated with recombinant human TGF-β1, insulin, or interferon-γ for the times as indicated in Fig. 1 (or data not shown). Total RNA was purified and reverse transcribed. RT-PCR was then performed with the primers specific to GDP-fucose (GDP-Fuc), CMP-sialic acid (CMP-SA), and UDP-GlcA/GalNAc transporters as well as β-actin. The results show a greatly increased mRNA level of GDP-fucose transporter during the time course of TGF-β1 induction (Fig.1A, panel 1). However, the mRNA level of UDP-GlcA/GalNAc transporter (panel 3) or β-actin (panel 4) was not changed. Interestingly, the expression of CMP-sialic acid transporter was slightly inhibited (Fig.1A, panel 2). None of these transporters responded to the stimulation of insulin or interferon-γ (data not shown). In agreement with the above results, qRT-PCR analysis (Fig. 1B) indicates a seven-fold increase of the mRNA level 6 h after TGF-β1 induction as compared with that at time 0 and the level maintained steady thereafter. The steady level may reflect an equilibrium state between the mRNA production and turnover. To detect the protein level change, whole cell lysate was prepared from the cells without or with TGF-β1 induction for 10 h and Western blotting was performed with rabbit anti-GDP-fucose transporter or β-actin antibodies. Consistent with the above results, the level of the GDP-fucose transporter protein was significantly increased, but not the β-actin control (Fig. 1C, left panel, compare lane 2 with lane 1). Quantitative analysis shows a two-fold increase of the GDP-fucose transporter protein level 10 h after TGF-β1 induction (Fig. 1C, right panel). The discrepancy of increases between the transcript and protein level may reflect a differential effect of the transcriptional and translational regulation on the GDP-fucose transporter gene expression. In general, our data presented here suggest that TGF-β1 stimulates the GDP-fucose transporter gene expression.

### The GDP-fucose Transporter Promoter Region between bp −330 and −268 is Critical for its Expression

**Figure 2:**
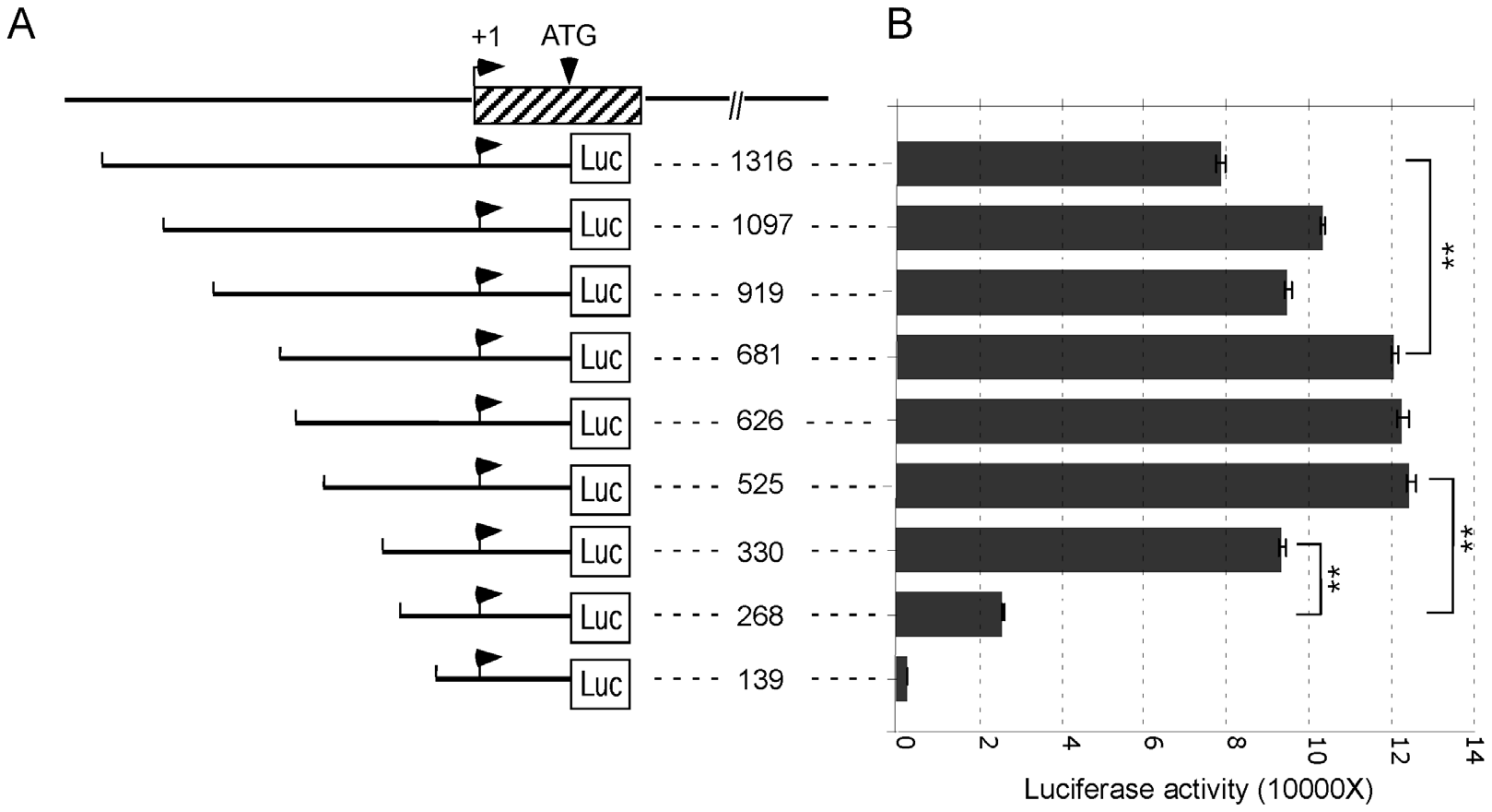
Promoter activity analyses of the GDP-fucose transporter gene. **A.** Schematic representation of the luciferase reporter constructs carrying serial deletions of the upstream sequences of the GDP-fucose transporter gene in pGL-3 basic vector. The numbers indicate the upstream sequences relative to the transcription initiation site. Luciferase start codon is indicated. **B.** Luciferase activity assays of the constructs described in A. The constructs containing various upstream promoter sequences as in A were transfected into HEK293 cells. After 24 h, the cells were harvested for whole cell lysates, which were used for luciferase activity analyses with Luciferase Assay Systems. The relative luciferase activity was measured by microplate luminometer. Error bars represent standard deviation from four independent experiments. **, p<0.01 (t-test).

To determine the cis-regulatory promoter elements responsible for the transcription of GDP-fucose transporter gene, we generated luciferase reporter constructs containing serial deletions of upstream sequences (from bp −1316 to −139) plus 453 bp sequence from +1 transcription initiation site. The start codon of luciferase is at the same position as that of the GDP-fucose transporter (Fig. 2A). We transfected the constructs into HEK293 cells, which have been shown to be efficient for protein expression. After 24 h, transfected cells were harvested for whole cell lysates, which were used for subsequent luciferase activity assays. The results show no major differences from serial deletions from bp −681 to −525 with respect to the luciferase activity (Fig. 2B), indicating no essential regulatory elements present in this region. However, deletion of a short fragment between bp −330 and −268 severely reduced the luciferase activity by ∼72%. Additional deletions from −139 to the start codon (ATG) did not affect luciferase activity, indicating that no internal elements important for its expression are located in this region (data not shown). We also found that the deletion of the region between bp −1316 and −681 resulted in a ∼54% increase of luciferase activity. The increase may be due to an inhibitory effect of another site within the deleted region of episomal DNA. Together, these results strongly suggest that the 62 bp promoter region (bp −330 to −268) of GDP-fucose transporter may contain the elements that are critical for its expression.

### The GDP-fucose Transporter Promoter Region between bp −330 and −268 Contains Dual Sp1-binding Motifs that are Essential for the Promoter Activity

**Figure 3:**
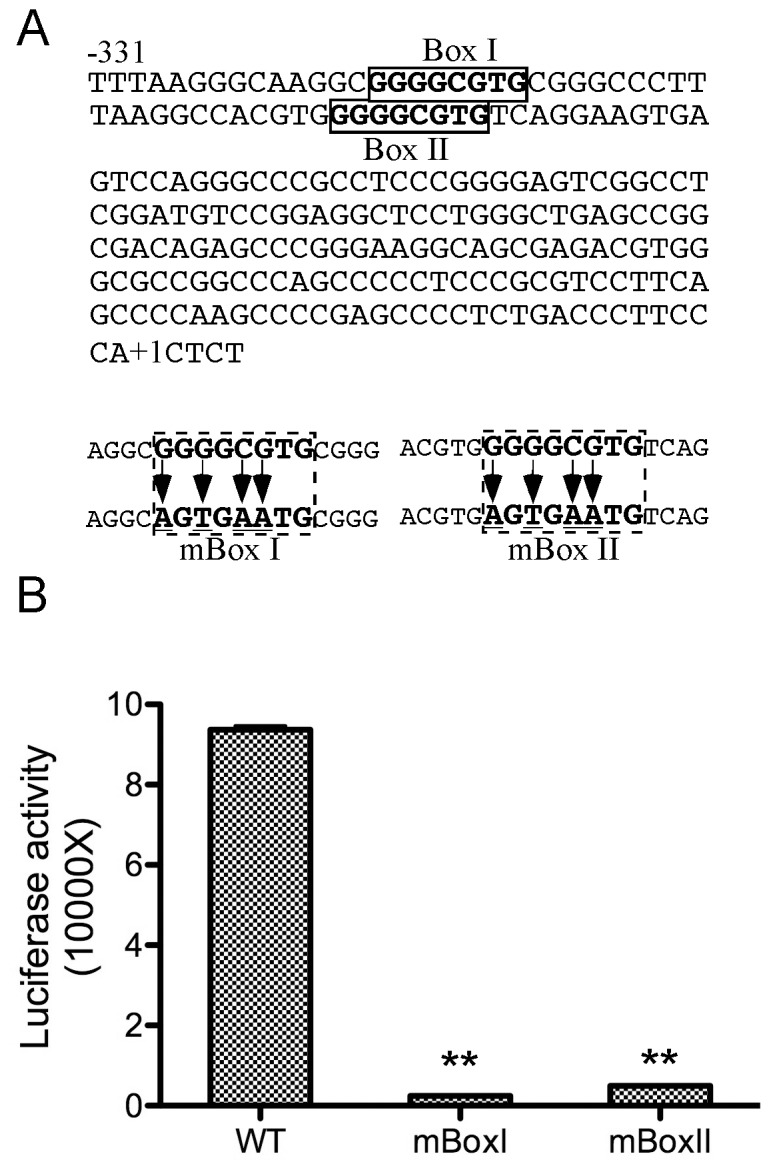
The GDP-fucose transporter promoter contains two identical GC-rich motifs that are essential for its expression. **A.** The GDP-fucose transporter promoter sequence from −331 to +5. The two GC-rich motifs are marked as Box I and II. Two mutated oligonucleotides (mBox I and II) with mutated nucleotides in the two boxes are shown at bottom. **B.** Mutagenesis analyses of the GDP-fucose transporter promoter GC-rich motifs. The construct containing 330 bp upstream sequence (WT) of the transporter promoter as in Fig. 2A and two similar constructs but with the mutated box I or II (mBox I or II) as in A were transfected into HEK293 cells and whole cell lysates were prepared and assayed for luciferase activity as in Fig. 2B. **, p<0.01 (t-test).

To determine the possible elements that might serve as binding sites for the GDP-fucose transporter gene-specific transcription factors, we performed sequence analyses of the promoter region between bp −330 and −268. We found that the 62 bp segment contains two identical GC-rich octamer motifs, GGGGCGTG (Fig. 3A), which are highly similar to the Sp1-binding site [24]. To obtain a direct evidence for the activity of the Sp1-binding motifs, we generated luciferase reporter constructs under the control of the WT 330 bp upstream sequence as shown in Fig. 2, and the same sequence but with four point mutations in GC-rich motif I or II (mBox I or mBox II, respectively) (Fig. 3A). The constructs were transfected into HEK293 cells and whole cell lysates were prepared for the luciferase activity assays as described above. The results show that the mutations in either GC-rich motif I or II severely affected the luciferase activity (>90% decrease) (Fig. 3B), indicating that the GC-rich motifs are essential for the transcription.

**Figure 4:**
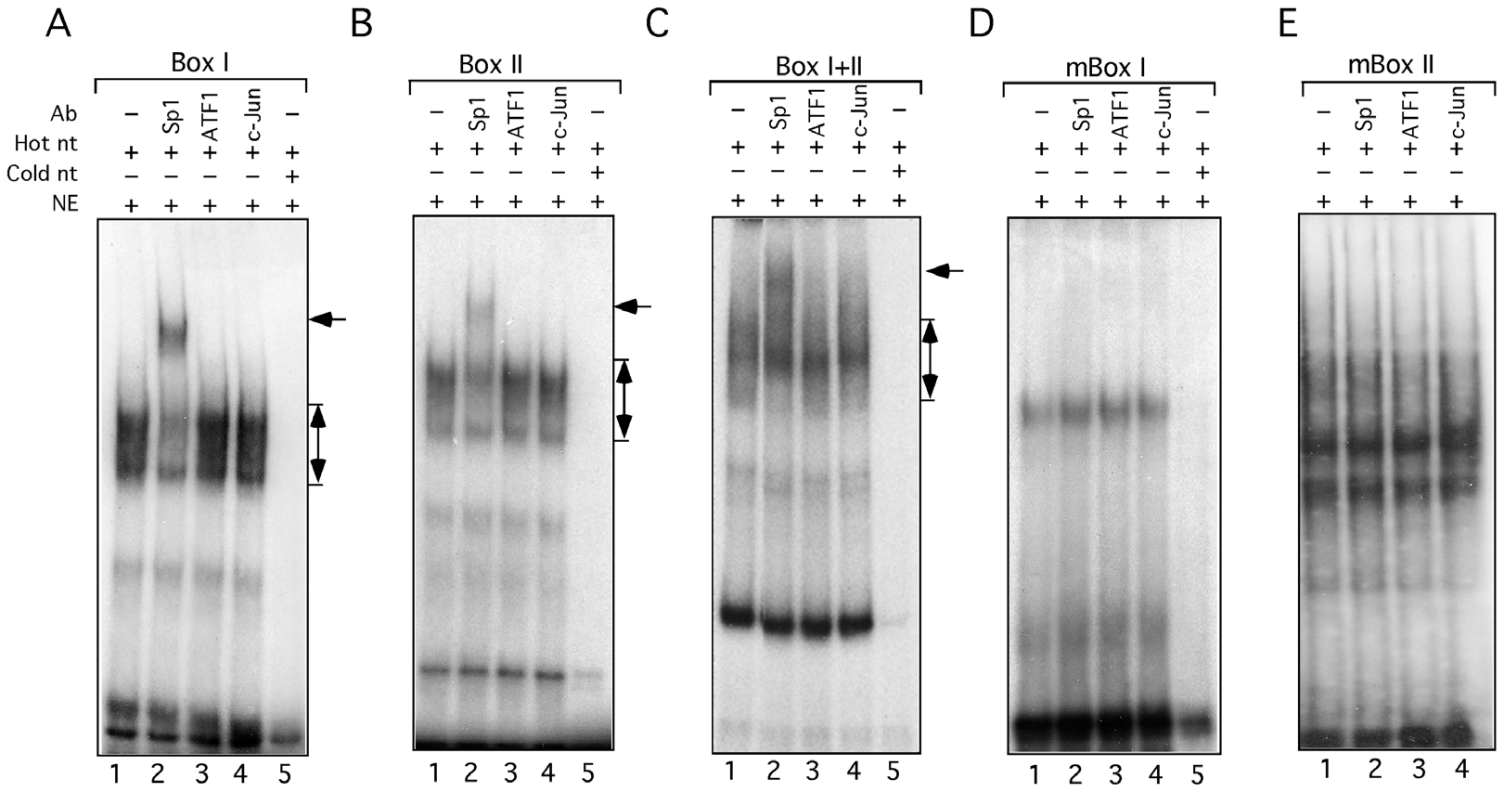
Transcription factor Sp1 specifically binds to the GC-rich motifs *in vitro*. Double strand oligonucleotides containing Box I (A), Box II (B), Box I+II (C), mutated Box I (D) or Box II (E) as in Fig. 3A were used for *in vitro* gel mobility shift assay. The components in the binding reactions including the labeled, unlabeled oligonucleotides, antibodies and nuclear extracts are indicated on top. The specific complex and super shift are indicated.

To test the GC-rich motifs for Sp1 binding, we generated three double strand oligonucleotides, which contain either box I, II or I+II, as well as two mutated oligonucleotides with four point mutations in box I or II, the same as shown in Fig. 3A. The oligonucleotides were labeled with [γ^32^P]ATP and subsequently used for gel mobility shift assays. Following incubation of the labeled oligonucleotides with HeLa nuclear extract, anti-Sp1, -ATF1, or -c-Jun antibodies were added for binding to endogenous nuclear proteins. The reaction mixtures were resolved on a native polyacrylamide gel. The results show that the double strand oligonucleotides of box I, II or I+II migrated in a broad range of molecular weights (Fig. 4A, B, and C, lanes 1–4), indicating that the oligonucleotides indeed formed heterologous complexes with nuclear proteins. Interestingly, addition of the antibody against Sp1 effectively induced a super shift (Fig. 4A, B, and C, lanes 2), indicating that Sp1 indeed participated in the formation of the complexes. In comparison, addition of the antibodies against ATF1 or c-Jun had no effect on the shifting (Fig. 4A, B, and C, lanes 3 and 4), suggesting that they were not present in the complexes.

To further confirm the binding specificity, we performed the gel mobility shift assays with the two mutated oligonucleotides as described above. We expected that the mutations would disrupt the interaction of Sp1 with the GC-rich motifs. The results show that the super shift from the WT oligonucleotide was not observed when either the mutant box I or II oligonucleotide was used (Fig. 4D and E, lanes 2), suggesting that the mutations indeed disrupted the interaction of Sp1with the GC-rich motifs.

### Sp1 and Smad2 Interact with the Promoter Region of GDP-fucose Transporter

**Figure 5:**
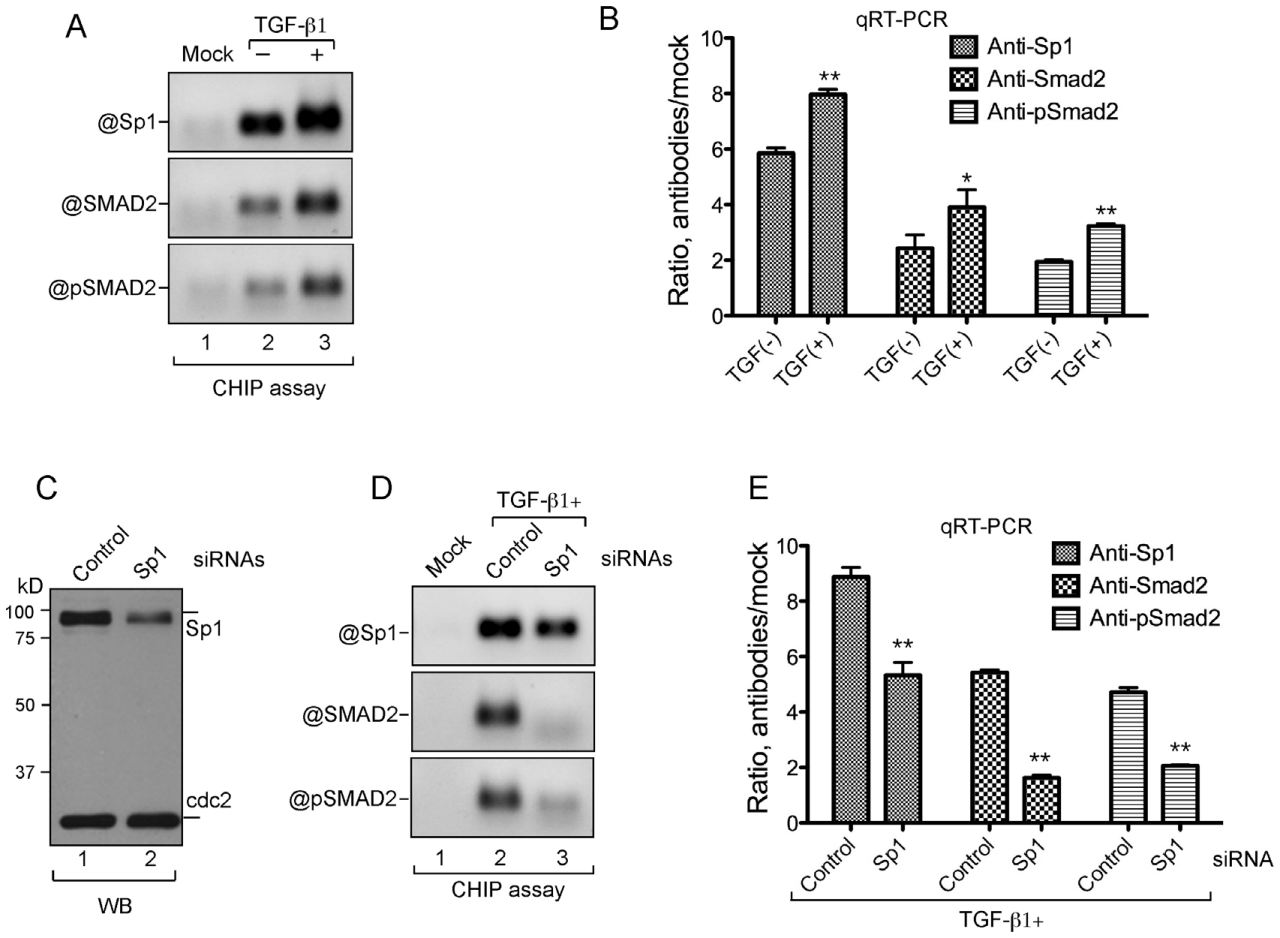
Sp1 and Smad2 are specifically associated with the GDP-fucose transporter promoter. **A.** and **B.** ChIP analyses of the interactions of Sp1 and Smad2 with the GDP-Fuc transporter promoter upon TGF-β1 stimulation. HeLa cells were serum-starved and then incubated with human TGF-β1 for 8 h. Extracts for ChIP assays were prepared from the cells treated without (lane 2) or with (lanes 1 and 3) TGF-β1. ChIP was carried out with rabbit immunoglobulin (mock) (lane 1), anti-Sp1 (top), -Smad2 (middle) or -pSmad2 (bottom) antibodies. The resulting precipitates were amplified by PCR with the primers specific to the GDP-fucose transporter promoter region. The PCR products were analyzed on a 1% agarose gel (A). qPCR was performed with the precipitated DNA from the ChIP assay as above (B). Error bars represent standard deviation from three replicates. *, p<0.05; **, p<0.01 (t-test). **C.** Western analyses of the down-regulation of Sp1 expression. HeLa cells were transfected with a control (lane 1) or Sp1-specific (lane 2) siRNAs. Whole cell lysate was prepared and analyzed by Western blotting. The same blot was probed with anti-Sp1 and cdc2 antibodies. Sp1 and cdc2 are indicated. **D** and **E.** The association of Smad2 with the GDP-fucose transporter promoter is Sp1 dependent. HeLa cells were transfected with control (lane 2) or Sp1-specific (lane 3) siRNAs followed by TGF-β1 stimulation. ChIP PCR (D) and qPCR (E) were performed and analyzed as in A and B.

Previous study showed that the TGF-β1 signaling activates Smad2 by phosphorylating its C-terminus and the phosphorylated Smad2 in turn interacts with Sp1 and enhances Sp1 DNA binding and its transcriptional activity [25]. To investigate whether Sp1 and Smad2 are assembled at the GDP-fucose transporter promoter upon induction with TGF-β1, we performed ChIP assays. Serum-starved HeLa cells were incubated without or with TGF-β1 (Fig. 5A, lanes 2 and 3, respectively). The cells were then treated with formaldehyde for protein-DNA crosslink. ChIP extracts were prepared and used for ChIP assays with the antibodies specific to Sp1, Smad2 or pSmad2 at serines 465/467. The precipitated DNA was used for end-point PCR with primers complementary to the GDP-fucose transporter promoter region covering the conserved Sp1-binding motifs as shown in Fig. 3A. Sp1, Smad2 and pSmad2 were found to interact with the promoter region without TGF-β1 induction (Fig. 5A, lanes 2), which may reflect the basal binding activity of these factors. Stimulation with TGF-β1 significantly increased the interaction of Sp1 with the promoter (top, lane 3), and the increased interaction was consistent with the elevated association of Smad2 and pSmad2 with the promoter (middle and bottom, respectively, lanes 3). The precipitated DNA from the ChIP assays was also used for qPCR analyses, and the results (Fig. 5B) show that the TGF-β1 induction increased the binding of Sp1, Smad2 and pSmad2 to the promoter region by ∼36%, ∼60% and ∼66%, respectively, as compared with the controls without TGF-β1. These results indicate that Sp1 and Smad2 are indeed assembled at the promoter region of the GDP-fucose transporter gene in response to TGF-β1 stimulation.

To further validate the binding specificity of Sp1 and Smad2 to the promoter region, we tested whether down-regulation of Sp1 expression would reduce or abolish their associations by using Sp1-specific siRNA–based ChIP assays. To confirm the siRNA effect on silencing Sp1 expression, whole cell lysates of HeLa cells transfected with the Sp1-specific or control siRNAs, were subjected to Western blotting analysis with anti-Sp1 and -cdc2 antibodies. The results show that Sp1 siRNA effectively reduced the Sp1 expression but not the cdc2 control (Fig. 5C, compare lane 2 with lane 1). Subsequently, we prepared the ChIP extracts from the cells transfected with Sp1 or control siRNAs followed by TGF-β1 induction. The results from ChIP assays show that the siRNA-mediated down-regulation of Sp1 significantly reduced the binding of Sp1 and Smad2/pSmad2 to the GDP-fucose transporter promoter (Fig. 5D, compare lane 3 with lane 2). We also performed qPCR by using the precipitated ChIP DNA to quantify the reduction. We found that, as compared with the controls, down-regulation of Sp1 reduced ∼40% of Sp1, ∼70% of Smad2 and ∼56% of pSmad2 binding to the promoter region (Fig. 5E). The discrepancy of the increases and reductions between Sp1 and Smad2 might be due to the fact that Sp1 has basal binding activity to the promoter, while Smad2 binding to the promoter is mainly dependent on the stimulation of TGF-β1. Therefore, the binding of Smad2 was more sensitive to the TGF-β1 stimulation leading to greater binding increase or decrease as shown in Fig. 5A, B, D, and E. Together, these results highlight that TGF-β1 activates the Smad2-containing complex, which in turn interacts with Sp1 and enhances its binding to the GDP-fucose transporter promoter.

### TGF-β1 Induces the GDP-fucose Transporter Expression in an Sp1-dependent Manner

**Figure 6:**
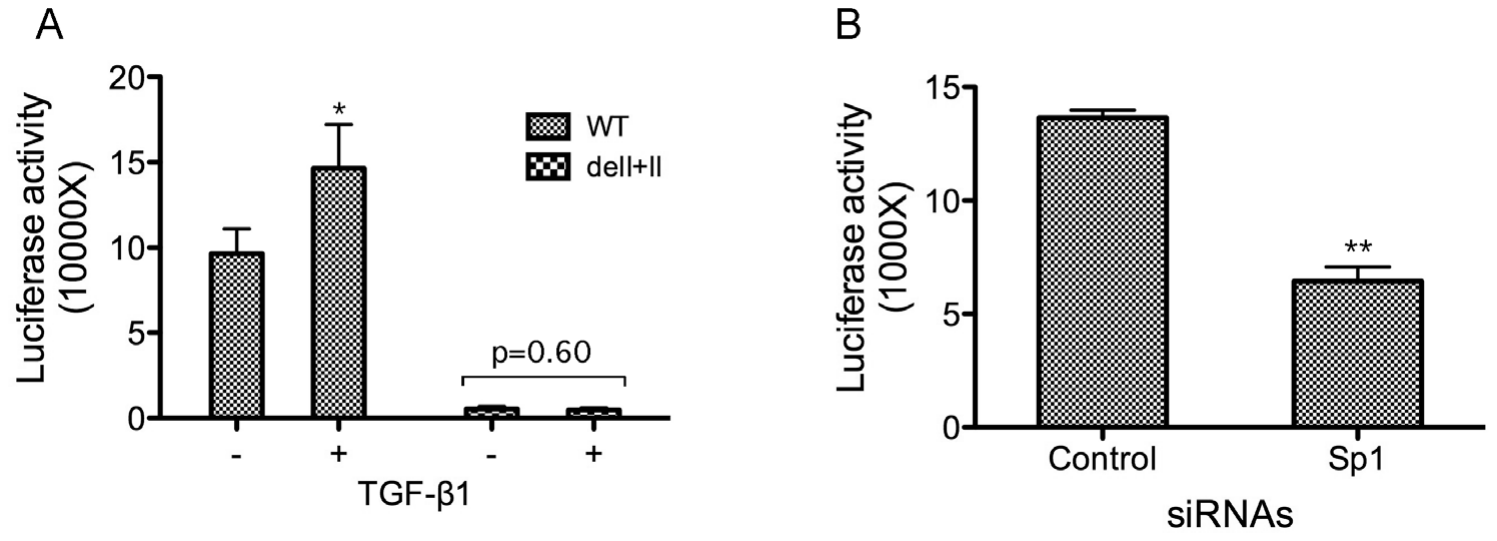
TGF-β1 stimulates the GDP-fucose transporter expression in an Sp1-dependent manner. **A.** Luciferase reporter constructs (in pGL-3 basic vector) carrying upstream sequences of 268 (delI+II) or 626 bp were transfected into HEK293 cells. The cells were first serum-starved, and then incubated without (−) or with (+) TGF-β1. The luciferase activity was measured as in Fig. 2B. *, p<0.05 (t-test). **B.** Down-regulation of Sp1 reduced the expression of the GDP-fucose transporter. HEK293 cells were transfected with a control or Sp1-specific siRNAs as in Fig. 5A. The siRNA-transfected cells were then transfected with a similar construct containing the GC-rich motifs as in A. The luciferase activity was measured as in A. The error bars represent standard deviations from three replicates. **, p<0.01 (t-test).

To obtain the gene-specific activity upon induction with TGF-β1, we performed transcriptional analyses by monitoring the luciferase activity under the control of the GDP-fucose transporter promoter. The luciferase reporter constructs carrying 268 bp (without two GC-rich motifs, delI+II) and 626 bp (with two GC-rich motifs) upstream sequences as shown in Fig. 2A and Fig. 3A, were transfected into HEK293 cells. Serum-starved cells were then incubated with or without TGF-β1. Whole cell lysates were prepared for the luciferase activity assays. The results show that TGF-β1 indeed increased the luciferase activity for the construct carrying 626 bp upstream sequence by ∼50% of the control, but not for the construct without two GC-rich motifs (delI+II) (Fig. 6A), suggesting that the GC-rich motifs are required for the response to TGF-β1 stimulation. To further validate that the transcription was Sp1 dependent, we again used the Sp1-specific siRNA to down-regulate its expression as described in Fig. 5A. A similar luciferase reporter construct containing GC-rich motifs as in Fig. 6A was used for the luciferase assays. The results show that the Sp1 down-regulation significantly reduced the luciferase activity by ∼50%, suggesting that Sp1 is crucial for the reporter gene expression (Fig. 6B). Overall, these results demonstrate that TGF-β1 induces the transcription of the GDP-fucose transporter gene and the transcription is dependent on the activation of Sp1.

## Discussion

NSTs have been identified and characterized in a wide range of species from mammals to lower eukaryotes such as yeast, fungi and parasitic protozoan, *Leishmania* reviewed in [7], and most recently we characterized four *Trypanosoma brucei* NSTs and demonstrated their essential role in glycosylation [26]. Various studies have shown that NSTs play a crucial role in growth, development and differentiation in mammals. However, little is known about their expression and transcriptional regulation. Here we employed a systematic approach to investigate the extracellular signal, intracellular pathway, promoter structure and gene-specific transcription factor for the GDP-fucose transporter gene. Among the growth factors examined, we found that TGF-β1 specifically induces the GDP-fucose transporter expression. We identified dual GC-rich octamer motifs in the transporter promoter region that are essential for its activity. We provided evidence that Sp1 specifically binds to the GC-rich motifs *in vitro*, and Sp1 coupled with Smad2 complex interacts with the motifs in response to the TGF-β1 induction *in vivo*. Collectively, this study provides detailed information about the transcriptional regulation for the GDP-fucose transporter gene and is also the first report of the transcriptional study for the NSTs identified so far in any eukaryotic organisms.

We showed that the transcription of GDP-fucose transporter is regulated by the TGF-β signaling pathway. TGF-β1 is a multifunctional cytokine and its signaling pathway regulates expression of numerous target genes and plays central roles in a variety of physiological events [27]. Its aberrant regulation is linked with various disease processes. Interestingly, fucosylation is essential for the TGF-β signaling, as the TGF-β1 receptor (type II) is modified with a fucose-containing core structure of *N*-glycan. The fucosylation is specifically catalyzed by α1, 6-fucosyltransferase Fut8. RNAi-mediated Fut8 silencing in human renal epithelial cells caused inactivation of TGF-β/Smad2/3 signaling [28]. Loss of core fucosylation in the Fut8-knockout mice affects several signaling pathways including TGF-β signaling. Consistent with this, the Fut8-knockout mice exhibited widespread growth defects including postnatal death, growth retardation and lung emphysema, which could be partially restored by injection of TGF-β1 [19], [29]. The regulation of glycosylation by glycosyltransferases in cellular signaling pathways may be a common phenomenon in various physiological events. For examples, a polypeptide GalNAc transferase, ppGalNAcT-1, was recently shown to modulate fibroblast growth factor (FGF) signaling through β1 integrin receptors [30]. GDP-fucose protein *O*-fucosyltransferase 1 (POFUT1), which is responsible for Notch receptor fucosylation, was shown to be essential for the normal Notch signaling [31].

Little is known about transcription regulation in glycosylation. Recently using a genome-wide association study, it was found that hepatocyte nuclear factor 1α (HNF1α), a transcription factor that regulates gene expression in liver and pancreas, also regulates the expression of key fucosyltransferase and fucose biosynthesis genes [32]. Accordingly, mutations from HNF1α have been shown to cause a significant change in plasma fucosylation profile [32], [33]. The findings from this study suggest that the transcription regulation of the GDP-fucose transporter may be important in the regulation of fucosylation as well as in the TGF-β signaling. Notably, the transport activity of NSTs is a rate-limiting step for the glycosylation in the Golgi apparatus [34]. As a result, the function of Fut8 is largely dependent on the availability of the GDP-fucose in the Golgi, which is provided by GDP-fucose transporter. Consistent with this, functional deficiency of GDP-fucose transporter caused impaired Notch signaling due to a fucosylation defect [9], [35]. Thus, it is likely that the regulation of the GDP-fucose transporter expression by TGF-β1 may also be able to provide a feedback to the TGF-β signaling *via* the modulation of TGF-β receptor fucosylation by Fut8.

This study demonstrated that the Sp1-binding motifs are essential for the GDP-fucose transporter gene expression. As compared with other well-defined promoters for house keeping genes [36], the GDP-fucose transporter core promoter has its unique features, e.g., the core promoter region is highly GC-rich and lacks AT-rich sequences and a TATA box in the promoter region close to the transcription initiation site, indicating that this transporter gene possesses a TATA-less core promoter structure [37], [38]. It was shown that in the TATA-less promoter, the initiator is a powerful element, which is the functional analogue to the TATA box.

The GDP-fucose transporter promoter indeed possesses a well-conserved initiator sequence, CCCA^+1^CTCT, in the defined core promoter region, which matches the initiator consensus sequence (PyPyCA^+1^NT/APyPy) [37]. A variety of the basal transcription factors are able to recognize the initiator sequence. TFIID can bind to the initiator element in a sequence-specific manner [39]–[43]. A purified RNA polymerase II complex containing TBP, TFIIB, and TFIIF, can recognize the initiator and mediate transcription initiation [44], [45]. In addition, the initiator may also serve as a binding site for the initiator-binding proteins such as TFII-I and Ying Yang 1 protein (YY1), which in turn facilitate the assembly of the basal transcriptional machinery [46], [47]. However, it should be noted that the binding of YY1 to the initiator of human DNA polymerase-β was not essential for the transcriptional complex assembly and not correlated with transcription activity [45]. Thus for the TATA-less promoters, the initiator sequence and the factors from the basal transcription machinery such as TFIID are important determinants for the transcription initiation complex formation. The initiator-binding proteins may participate in the complex assembly at a specific subset of promoters.

The GDP-fucose transporter expression is upregulated by the TGF-β signaling pathway *via* activation of Sp1, which is one of the well-established signaling pathways. The regulation of TGF-β signaling pathway is complex, because the pathway is embedded in a broad range of protein-protein interaction network [48], [49]. The key players that transduce TGF-β signals, are the intracellular effectors, Smads. The differential recruitment of Smad factors actually defines the specificity of the TGF-β signaling for gene expression. TGF-β1 stimulates phosphorylation of Smad2 and/or Smad3 at the conserved C-terminal SSXS motif. The phosphorylation induces Smad2 and/or Smad3 to form a complex with the co-Smad, Smad4, which then translocates into the nucleus, where the complex regulates the transcription of TGF-β1 target genes by activating various transcription factors [27]. In this study we provided the evidence that the phosphorylated Smad2 and Sp1 were specifically associated with the promoter region containing the Sp1-binding motifs upon the stimulation of TGF-β1. Our results are consistent with the previous studies on the transcriptional regulation of the TGF-β1 target genes, whose activation is Smad- and Sp1-dependent. One of the examples is the transcription of p^15Ink4B^, a cyclin-dependent kinase inhibitor [25]. TGF-β1 stimulates the p^15Ink4B^ expression by inducing a complex assembly of Smad2, Smad3, Smad4, and Sp1 on its promoter. Mutations in the Sp1- or Smad-binding sequences significantly reduced the TGF-β responsiveness of the p^15Ink4B^ promoter [25]. Similar to our results, Smad2 associated with Sp1 was shown to enhance the Sp1 DNA binding and its transcriptional activity. Our studies on the transcriptional activation of the GDP-fucose transporter gene induced by TGF-β1 added another example to this canonical signaling pathway.

We previously showed that the GDP-fucose transporter is crucial for fucosylation, synthesis and secretion of a broad range of proteins [14]. TGF-β1 signaling itself was observed to be regulated by fucosylation, because its key component, TGF-β1 receptor, is fucosylated by Fut8 [19]. The finding from this study suggests a link between the transporter gene expression and the TGF-β signaling, which may be required for maintenance of its target gene expression at relatively constant levels under certain circumstances. Thus, it will be interesting to investigate the transcriptional regulation of other factors involved in fucosylation such as Fut8, whose transcription regulation remains unknown. In addition, unlike GDP-fucose transporter, our preliminary results showed that the gene expression of CMP-sialic acid and UDP-GlcA/GalNAc transporters did not respond to the induction of TGF-β1, suggesting a diversity of regulatory pathways for the mammalian NST expression. Therefore, investigation of the transcriptional regulation of other NSTs will deepen our understanding of biological roles of NSTs and their concomitant effect on glycosylation under normal physiological and pathological conditions.
